# Relationship between the practice of chemsex and taking PrEP

**DOI:** 10.1192/j.eurpsy.2024.728

**Published:** 2024-08-27

**Authors:** O. De Juan Viladegut, H. Andreu Gracia, L. Bueno Sanya, L. Olivier Mayorga, E. Cesari, J. I. Mena, I. Ochandiano, S. Salmeron, L. Miquel

**Affiliations:** ^1^Psychiatry and Psychology Department, Hospital Clínic de Barcelona, Institut Clínic de Neurociències, Barcelona, Spain

## Abstract

**Introduction:**

Chemsex refers to the use of drugs, typically stimulants and/or psychoactive substances, in a sexual context, often in the context of casual or group sex encounters. Currently, the practice of chemsex focuses on men who have sex with men (MSM). On the other hand, Pre-exposure prophylaxis (PrEP) is a biomedical method that has proven effective in preventing HIV transmission, particularly among individuals at a heightened risk, including those who engage in chemsex. MSM account for two thirds of new HIV cases in the US. It is estimated that in 70% of cases seroconversion occurs through “condomless anal sex” (CAS). According to the CDC, one in six MSM will be infected with HIV during their lifetime. The consumption of methamphetamine (MA) has been identified as the main driver of the practice of CAS, alteration of rectal immunological function and faster seroconversion. One in three new HIV infections have been associated with MA consumption. (Grov C *et al*. JAIDS 2020; 85 272-279).

**Objectives:**

The primary goal of this study is to describe the prevalence of chemsex engagement among PrEP users, delineate user characteristics and requirements, gain deeper insights into this phenomenon within the Barcelona region, and formulate customized strategies accordingly.

**Methods:**

This study conducts a literature review to explore the current correlation between engaging in chemsex and the utilization of PrEP. We identified research articles published between January 2020 and December 2022, that discussed the utilization of chemsex drugs prior to or during sexual activities. The findings were synthesised using a narrative approach and conceptualised using a behavioural analysis framework.

**Results:**

According to a recent cross-sectional study performed at Hospital Clínic de Barcelona, SUD among patients who are being followed-up in the outpatient clinic of PrEP was higher (89%) compared with other European regions such as England (38.5%) or Amsterdam (41%). Moreover, according to data collected in the EMIS 2017 survey, Barcelona is the city with the highest prevalence of chemsex in Spain. (De La Mora L *et al*. AIDS Beh. 2022; 26: 4055-4062).

**Conclusions:**

The frequency of chemsex practice among individuals using PrEP in Barcelona surpasses what has been observed in other groups. Nearly 25% of the participants express worries and a requirement for assistance regarding the management of drug use, matters associated with their sexuality, and sexually transmitted infections (STIs). MSM who suffers from substance use disorder may experience difficulty achieving effective daily oral PrEP adherence prevention levels that may serve as early indicators of increased risk of disengagement from PrEP care and discontinuation the PrEP. These results highlight the importance of adopting aninterdisciplinary approach that incorporates education about substances and the implementation of risk mitigation strategies within the context of riskier sexual behaviors.

**Disclosure of Interest:**

None Declared

